# Identification and Analysis of Antimicrobial Activities from a Model Moss *Ceratodon purpureus*

**DOI:** 10.3390/metabo13030350

**Published:** 2023-02-27

**Authors:** Ashley L. Dague, Lia R. Valeeva, Natalie M. McCann, Margarita R. Sharipova, Monica A. Valentovic, Lydia M. Bogomolnaya, Eugene V. Shakirov

**Affiliations:** 1 Department of Biomedical Sciences, Joan C. Edwards School of Medicine, Marshall University, Huntington, WV 25755, USA; 2 Department of Biological Sciences, College of Science, Marshall University, Huntington, WV 25701, USA; 3 Institute of Fundamental Medicine and Biology, Kazan Federal University, Kazan 420008, Russia

**Keywords:** fire moss, bryophyte, antibacterial activity, exudate, moss, plant metabolite

## Abstract

The emergence of bacterial drug resistance is often viewed as the next great health crisis of our time. While more antimicrobial agents are urgently needed, very few new antibiotics are currently in the production pipeline. Here, we aim to identify and characterize novel antimicrobial natural products from a model dioicous moss, *Ceratodon purpureus*. We collected secreted moss exudate fractions from two *C. purpureus* strains, male R40 and female GG1. Exudates from the female *C. purpureus* strain GG1 did not exhibit inhibitory activity against any tested bacteria. However, exudates from the male moss strain R40 exhibited strong inhibitory properties against several species of Gram-positive bacteria, including *Staphylococcus aureus* and *Enterococcus faecium*, though they did not inhibit the growth of Gram-negative bacteria. Antibacterial activity levels in *C. purpureus* R40 exudates significantly increased over four weeks of moss cultivation in liquid culture. Size fractionation experiments indicated that the secreted bioactive compounds have a relatively low molecular weight of less than 1 kDa. Additionally, the R40 exudate compounds are thermostable and not sensitive to proteinase K treatment. Overall, our results suggest that the bioactive compounds present in *C. purpureus* R40 exudates can potentially add new options for treating infections caused by antibiotic-resistant Gram-positive bacteria.

## 1. Introduction

Bacterial infections lead to a number of human diseases and represent a leading cause of morbidity and mortality worldwide. A recent report by Antimicrobial Resistance Collaborators [1] indicated that the 33 most common bacterial pathogens caused up to 13.6% of all deaths in the world and 56.2% of all sepsis-related deaths in 2019. Among all bacterial pathogens, the Gram-positive *Staphylococcus aureus* was the leading bacterial cause of death in 135 countries and was also associated with the most deaths in individuals older than 15 years. Another Gram-positive pathogen, *Streptococcus pneumoniae,* was the bacterial species associated with the most deaths in children younger than 5 years. Overall, deaths associated with pathogenic bacteria ranked as the second leading cause of death globally in 2019 [1].

One of the main reasons bacterial infections continue to lead to high mortality throughout the world is the rapidly growing antibiotic resistance among many pathogens [2,3]. The emergence and spread of antibiotic resistance among pathogenic bacteria is a disturbing trend recently identified by the World Health Organization (WHO) as one of the ten biggest health challenges of our time (http://sdg.iisd.org/news/who-identifies-top-health-challenges-begins-five-year-health-plan/) (accessed on 30 January 2023). In its 2019 report, the Centers of Disease Control and Prevention categorized different antibiotic-resistant bacteria as urgent, serious, and concerning pathogens (https://www.cdc.gov/drugresistance/biggest-threats.html) (accessed on 30 January 2023). Among these pathogens, several multidrug-resistant (MDR) Gram-positive bacteria are considered major healthcare problems [4]. These include methicillin-resistant *Staphylococcus aureus* (MRSA), which in 2017 caused 323,700 estimated cases in hospitalized patients and 10,600 estimated deaths, with USD 1.7 Billion estimated attributable healthcare costs. Also on the rise are infections caused by vancomycin-resistant *Enterococcus faecium* (VRE), which caused 54,500 estimated cases in hospitalized patients and 5400 estimated deaths in 2017. Another Gram-positive pathogen of concern is erythromycin-resistant Group A Streptococcus (GAS), which caused approximately 1 to 2.6 million cases of strep throat and invasive infections (cellulitis, pneumonia, flesh-eating infections, and sepsis), and up to 1900 deaths. These and other pathogens are becoming increasingly resistant to available antibiotics, raising concerns that the last remaining drugs to treat Gram-positive bacterial infections may become less effective. Thus, the rise of antimicrobial resistance demands increased efforts to discover new antibiotics.

Antibacterial drug development based on previously characterized chemical scaffolds is arguably reaching its technical limitations [5]. On the other hand, natural products produced by previously largely untapped groups of organisms, such as plants, could be a powerful source of new antimicrobials with potentially novel chemical structures [6]. Specifically, plant-derived natural antimicrobials have been historically used in traditional folk medicine, but not very commonly utilized by the modern pharmaceutical industry. Indeed, plants are known to produce a variety of potent secondary metabolites with important medical benefits, including well-characterized compounds such as acetylsalicylic acid, artemisinin, and paclitaxel [7]. In particular, members of the Bryophyte group (mosses, liverworts, and hornworts) are known to produce a plethora of biologically active secondary metabolites [8]. To date, a number of different natural compounds with antimicrobial, antioxidant, and ultraviolet-screening properties have been detected in bryophytes [9,10,11]. Specifically, metabolites from several mosses were shown to effectively inhibit the growth of Gram-negative or Gram-positive bacteria [12,13]. However, detailed studies aimed at chemical characterization of antibacterial secondary metabolites from Bryophytes are still lacking, especially in model moss species.

Even less is known about antibacterial properties of extracellular metabolites secreted by plants, many of which play a crucial role in communication with the root microbiota, including important herbivores and bacterial pathogens [14,15,16]. The model liverwort *Marchantia polymorpha*, for example, secretes specialized “oil bodies” containing sesquiterpenes and cyclic bisbibenzyls, which serve as a defense against arthropod herbivores [17,18]. The model moss *Physcomitrium patens* secretes a number of extracellular compounds and peptides with antibacterial properties [19,20]. For potential future biotechnological applications, such secreted bioactive metabolites may offer several technical advantages over intracellular compounds, as they are usually water-soluble and can be easier to biochemically isolate and purify.

*Ceratodon purpureus* (also known as the “fire moss”) is a biogeographically diverse model moss species [21], known for its ability to grow on soils contaminated with heavy metals, in areas of high UV radiation, under salt and cold stress, and even in extremely cold conditions of Antarctica. *C. purpureus* is dioicous and harbors gene-rich UV sex chromosomes, enabling researchers to study how the presence of sex-linked genes affects plant sexual development and metabolism [22,23]. Recent data point to intriguing differences between male and female strains of *C. purpureus* in the amount and composition of produced volatile organic compounds (VOCs) that can affect the behavior of soil microarthropods and attract them to aid in moss fertilization processes [23,24]. Overall, due to its small size, ease of laboratory cultivation, sequenced and annotated genome, and rapidly developing metabolomics resources, *C. purpureus* is currently becoming a model organism of choice for many types of plant research [22,25,26].

Here, we aim to discover and characterize antibacterial metabolites secreted by the model moss *C. purpureus*. We detected an unexpected sexual dimorphism in the production of *C. purpureus* antibacterial metabolites: exudates from the male moss strain R40 contain potent antibacterial activity, while exudates from the female strain GG1 do not. Interestingly, R40 exudates are effective against several species of Gram-positive bacteria, but do not inhibit the growth of Gram-negative bacterial species. The mode of action of R40 exudate components appears to be bactericidal with a minimum inhibitory concentration of 6.25 mg/mL. Exudate stability and light sensitivity experiments indicate that secreted metabolites are relatively stable after boiling or multiple freezing/thawing cycles, in different light conditions and after treatment with Proteinase K, suggesting that the bioactive metabolites likely do not have a polypeptide structure. Furthermore, size fractionation experiments indicate that the metabolite with antibacterial activity has a low molecular weight of below 1 kDa. Taken together, our data suggest that *C. purpureus* antibacterial compounds represent secreted small metabolites with a bactericidal mode of action against Gram-positive bacteria. Overall, further characterization of bioactive *C. purpureus* compounds may lead to the development of novel antibacterial therapeutics.

## 2. Materials and Methods

### 2.1. Moss Strains, Growth Conditions and Exudate Collection

*C. purpureus* R40 (male) and GG1 (female) strains (a gift from Dr. Stuart McDaniel, University of Florida) were propagated on Petri plates with BCD nutrient agar medium as described before [20,27]. Moss was propagated weekly by homogenizing tissue with the IKA Ultra-Turrax T10 basic tissue dispenser, followed by plating the homogenized samples onto cellophane disks on top of solid BCD nutrient medium in Petri dishes. Moss plates were grown in a plant growth chamber (Model 7300, Caron Products) at 22 °C, 65% humidity, 880 lux light intensity, and 12/12 h light/dark conditions.

For secreted metabolite analysis, *C. purpureus* strains were grown in 250 mL flasks with 100 mL of the BCD liquid nutrient medium on the orbital shaker (at 150 rpm) at 22 °C, 65% humidity, 880 lux light intensity, and 12/12 h light/dark conditions. Exudate was collected and processed as described previously [20]. Frozen exudate samples were dried using a lyophilizer (model 7382021, Labconco, Kansas City, MO, USA) and stored at −80 °C until needed. For experimental analysis, dry samples were weighed and dissolved in sterile BCD medium with CaCl_2_ in the final concentration of 100 mg/mL.

### 2.2. Tests for Antibacterial Activity

Antimicrobial activity of moss exudates was analyzed against Gram-positive (*Staphylococcus aureus* ATCC 25923, *Streptococcus pyogenes* ATCC 12344, and *Enterococcus faecium* ATCC 35667) and Gram-negative bacteria (*Serratia marcescens* SM6, *Salmonella enterica ser*. Typhimurium ATCC14028s). Gram-positive bacterial strains were grown in Tryptic Soy medium and Gram-negative bacterial strains were grown in LB medium.

Disk-Diffusion assays (DDM). Bacterial growth inhibition was tested first using the qualitative DDM method in LB or Tryptic Soy agar according to CLSI guidelines (www.clsi.org, accessed on 1 July 2022). Bacteria in liquid cultures were grown overnight (ON) at 35 °C and shaken at 200 rpm using an orbital incubator shaker (Excella E24 Incubator Shaker Series, New Brunswick Scientific). The bacterial inoculum (CFU (colony-forming units) = 1 × 10^7^/plate) was prepared by dilution of 25 µL of ON culture in 5 mL of top agar (LB broth powder 25 g/L, 0.7% agar or Tryptic Soy broth powder 30 g/L, 0.7% agar), stirred and poured onto a Petri dish containing 20 mL of LB or Tryptic Soy agar. Next, 17.5 mg of moss exudate was dissolved in BCD medium and added onto sterile Whatman disks (disk diameter = 7 mm). Disks soaked with the same amount of sterile liquid BCD medium were used as the negative control and disks soaked with carbenicillin or chloramphenicol antibiotics were used as the positive control, as described previously [20]. Disks were placed on top of inoculated plates and incubated at 35 °C for 18 h. The diameter of the bacterial growth inhibition zone (halo) around each disk was then measured in mm and plotted. All experiments were carried out in triplicate on at least two separate occasions.

A broth microdilution method was used as a quantitative assay to determine the Minimal Inhibitory Concentration (MIC) of the moss exudate metabolites in a 96-well microtiter plate using the Synergy HTX BioTek plate reader spectrophotometer as described previously [20,28]. Moss exudates were added to bacterial cultures in twofold serial dilutions (100, 50, 25, 12.5, 6.25 mg/mL or less, depending on the experiment). The 96-well plate was sealed with a Breathe-Easy membrane (Diversified Biotech) to minimize evaporation, and the plate was incubated and continuously shaken at 35 °C for 18 h, with the optical density of the culture measured at 600 nm (OD600) every 15 min. MH broth with bacteria in the absence of exudates was used as the negative control, while a range of carbenicillin and chloramphenicol dilutions were used as the positive controls. MIC was defined as the lowest concentration of an antimicrobial agent that inhibits the visible growth of a bacterial culture. Each experiment was performed in four biological and three technical replicates.

### 2.3. Evaluation of Minimal Bactericidal Concentration

To determine MBC, bacterial cultures were prepared for MIC testing as described previously [20,28], and the inoculum aliquot was serially diluted and plated for CFU determination. The remaining bacterial cultures were grown overnight in the presence of different exudate concentrations in a 96-well microtiter plate using the Synergy HTX BioTek plate reader as described above for the MIC assays. After completion of this step, 50 µL of bacterial culture from each well (starting at the MIC concentration and higher) was plated onto LB plates and incubated overnight at 35 °C for 18 h. The number of growing bacterial colonies on each plate were counted to determine the CFU. The MBC was defined as the lowest concentration that demonstrates a 99.9% reduction in CFU compared to the inoculum [29].

### 2.4. Size Fractionation of Extracellular Metabolites

The Pall Corporation Macrosep Advance Centrifugal Devices (10 kDa, 3 kDa, and 1 kDa molecular weight cutoffs) were used to separate metabolite exudate components by molecular weight. Fractionation was performed following manufacturer instructions at 4 °C. Fractions of <1 kDa, 1–3 kDa, 3–10 kDa, and >10 kDa were collected, dried down, and analyzed in DDM or MIC assays.

### 2.5. Exudate Metabolite Stability and Sensitivity Tests

To determine light sensitivity, crude dried moss exudates were dissolved in BCD nutrient medium and either exposed to white light in a transparent microcentrifuge tube or kept in a similar microcentrifuge tube but covered with foil for 3 h at room temperature, followed by the MIC assay to determine residual activity. For thermostability assays, exudates were first subjected to size fractionation, and active <1 kDa fraction was subjected to two temperature treatments: boiling for ten minutes or repetitive thawing at 37 °C in a water bath and flash freezing in liquid nitrogen (three times total), followed by the MIC analysis to determine residual antibacterial activity. For Proteinase K sensitivity assay, 250 µg of active <1 kDa fraction was incubated with 32 µL (20 mg/mL) of proteinase K for 3 h at 37 °C, followed by the MIC analysis.

### 2.6. Data Analysis

Data are reported as mean ± standard deviation. Statistical significance was determined via an unpaired *t*-test with Welch’s correction; *p* < 0.05. Analysis was performed using GraphPad Prism v.9.5.0.

## 3. Results

### 3.1. Identification of Antibacterial Activity in C. purpureus exudates

**Figure 1:**
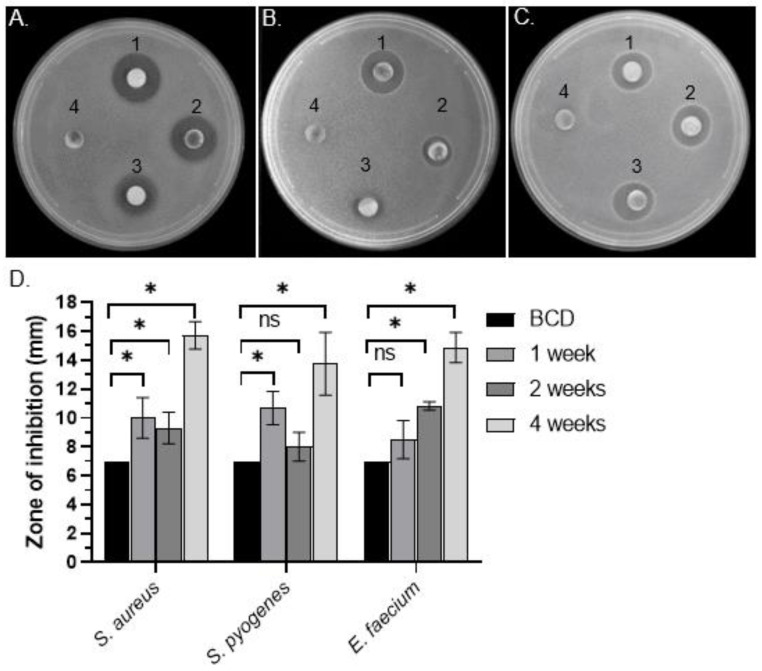
Bacterial growth inhibitory activity of extracellular metabolites of *C. purpureus* strains. (**A**–**C**) Representative pictures of qualitative DDM assays with filter disks soaked with exudates from four-week-old *C. purpureus* R40 strain and placed on top of *S. aureus* ATCC25923 (**A**), *S. pyogenes* ATCC12344 (**B**), and *E. faecium* ATCC35667 (**C**) bacterial lawns. (1–3) Disk with *C. purpureus* exudates; (4) negative control disks containing only BCD medium. (**D**) Diameter of *S. aureus* growth inhibition area (halo) around each cellulose disk containing secreted *C. purpureus* metabolites after one, two, and four weeks of moss growth was measured and plotted. Data represent the means from at least 3 independent experiments and a standard deviation. The asterisks indicate significance in an unpaired *t*-test; *—statistical significance *p* ≤ 0.05; ns—not significant.

To test for the presence of antibacterial activity in *C. purpureus* exudates, R40 (male) and GG1 (female) moss strains were grown in BCD liquid cultures and their exudates were collected after 1, 2, and 4 weeks of growth. DDM assays detected distinct halos around filter disks containing R40 exudates when tested against Gram-positive *S. aureus* (Figure 1A), *S. pyogenes* (Figure 1B), and *E. faecium* (Figure 1C) bacteria, but not against Gram-negative *S. marcescens* or *S. enterica* (Table 1). Interestingly, significant levels of antibacterial activity were detected in R40 exudates after as little as 1 week of moss growth in liquid culture, with the highest activity detected at 4 weeks of growth, when the experiment was stopped (Figure 1D). These data suggest that moss cells continuously secreted bioactive metabolite/s when grown in liquid culture. Surprisingly, however, exudates of the *C. purpureus* female strain GG1 did not show antimicrobial activity in DDM assays against either Gram-positive or Gram-negative bacteria (Table 1). Collectively, these data indicate that the *C. purpureus* R40 strain produces extracellular metabolites with high inhibitory activity against a number of Gram-positive bacteria and also provide an intriguing example of sexual dimorphism in the production or exudation of antibacterial moss metabolites.

### 3.2. Quantitative Analysis of Antimicrobial Activity from C. purpureus R40 Exudate

**Figure 2:**
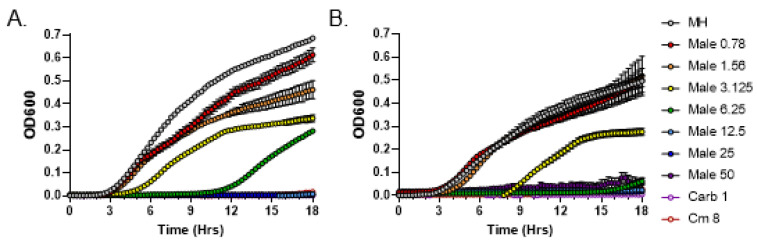
Broth microdilution method to determine the Minimal Inhibitory Concentration (MIC) of metabolites present in *C. purpureus* R40 exudates. Exudates from two-week-old (**A**) and four-week-old (**B**) *C. purpureus* R40 strains were tested in MIC assays against *S. aureus* ATCC25923. Growth curve of *S. aureus* cells was monitored in the presence of 0.781, 1.56, 3.125, 6.25, 12.5, 25, and 50 mg/mL of exudate solution. MH—negative control, no exudate added. Carbenicillin (Carb, 1 µg/mL) and chloramphenicol (Cm, 8 µg/mL) treatments were used as positive controls for *S. aureus* growth inhibition.

To quantitatively measure the minimum inhibitory concentration (MIC) of *C. purpureus* exudates following the CLSI guidelines, we employed a broth microdilution method. Previous data indicated that BCD moss growth medium alone (negative control) does not inhibit the growth of *S. aureus* [20]. We serially diluted exudates from the male R40 strain to 50, 25, 12.5, 6.25, 3.125, 1.56, and 0.781 mg/mL concentrations, which were incubated with *S. aureus* cultures in MIC assays. Exudate from R40 strain grown for 2 weeks inhibited the growth of *S. aureus* at a concentration of 12.5 mg/mL (Figure 2A), while exudate from this strain grown for 4 weeks, as previously suggested by the DDM assays, had a stronger inhibitory activity and displayed a 2-fold lower MIC value of 6.25 mg/mL (Figure 2B). These data establish that longer growth times for the male R40 strain lead to higher antibacterial activity of its exudate.

**Figure 3:**
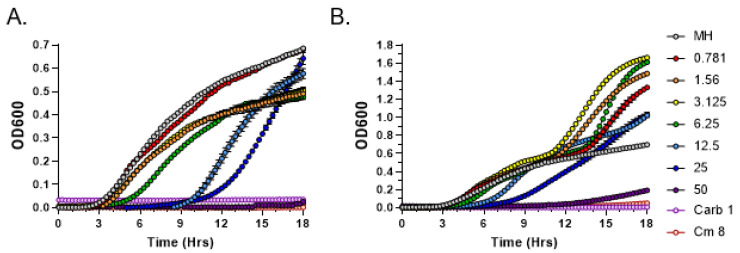
Broth microdilution method to determine the Minimal Inhibitory Concentration (MIC) of metabolites present in *C. purpureus* GG1 exudates. Exudates from two-week-old (**A**) and four-week-old (**B**) *C. purpureus* GG1 strains were tested in MIC assays against *S. aureus* ATCC25923. Growth curve of *S. aureus* cells was monitored in the presence of 0.781, 1.56, 3.125, 6.25, 12.5, 25, and 50 mg/mL of exudate solution. MH—negative control, no exudate added. Carbenicillin (Carb, 1 µg/mL) and chloramphenicol (Cm, 8 µg/mL) treatments were used as positive controls for *S. aureus* growth inhibition.

For comparison purposes, exudates from the female GG1 strain grown for 2 and 4 weeks were also tested in MIC assays. In both cases, bacterial growth inhibition was observed only at the highest exudate concentration of 50 mg/mL (Figure 3). Overall, these data confirm qualitative and quantitative differences in antibacterial potential between the male and female strains of *C. purpureus*.

**Figure 4:**
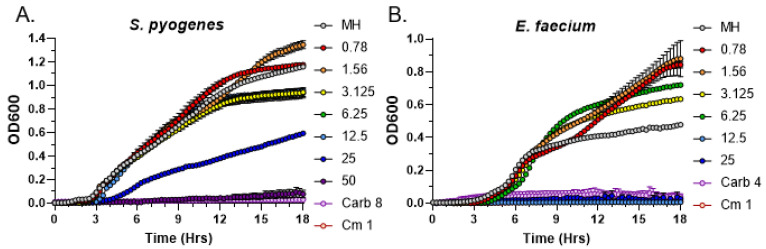
Analysis of the Minimal Inhibitory Concentration of *C. purpureus* R40 exudates against other Gram-positive bacteria. Exudates from four-week-old *C. purpureus* R40 strain were tested in MIC assays against *S. pyogenes* ATCC12344 (**A**) and *E. faecium* ATCC35667 (**B**). Growth curve of *S. pyogenes* and *E. faecium* cells was monitored in the presence of 0.781, 1.56, 3.125, 6.25, 12.5, 25, and 50 mg/mL of exudate solution. MH—negative control, no exudate added. Carbenicillin (Carb, 8 µg/mL or 4 µg/mL) and chloramphenicol (Cm, 1 µg/mL) treatments were used as positive controls for growth inhibition of *S. pyogenes* and *E. faecium*.

We next tested the most potent exudate from 4-week-old R40 strain in MIC assays against two other Gram-positive bacteria, *Streptococcus pyogenes* and *Enterococcus faecium*. While R40 exudates were indeed able to inhibit the growth of both bacteria, the MIC values were different. Specifically, the MIC value for *S. pyogenes* was 50 mg/mL (Figure 4A), while for *E. faecium* it was 4 times lower, 12.5 mg/mL (Figure 4B). These data indicate that both *S. aureus* and *E. faecium* are much more sensitive than *S. pyogenes* to the bioactive metabolites present in *C. purpureus* R40 exudates. Nevertheless, our quantitative MIC data confirm that antimicrobial compounds secreted by the *C. purpureus* R40 moss strain are effective against a spectrum of Gram-positive bacteria.

### 3.3. C. purpureus R40 Exudates Display Bactericidal Mode of Action

In general, antibiotics are divided into two groups based on whether they kill bacteria (bactericidal) or suppress bacterial growth (bacteriostatic). While both antibiotic types are often equally efficient in terms of clinical outcomes [30], understanding their mode of action is the first step in establishing their efficacy, especially since various antibiotics can be bacteriostatic for some pathogens and bactericidal for others. To test the mode of action for moss exudates, we followed the standard protocol for determining the ratio of minimum inhibitory concentration (MIC, the concentration that inhibits visible bacterial growth at 24 h of growth) to the minimum bactericidal concentration (MBC, the concentration of a compound that results in a 1000-fold reduction in bacterial CFU (colony-forming units) at 24 h of growth in the same specific conditions [31]. We monitored *S. aureus* growth dynamics for several *C. purpureus* exudate concentrations starting with 6.25 mg/mL and then plated serial dilutions of the growth cultures on LB plates to count the number of surviving colonies. Interestingly, no *S. aureus* colonies were detected in any plates containing bacterial cultures following incubation with all tested *C. purpureus* R40 exudate concentrations. Given that the calculated MIC value for *S. aureus* is 6.25 mg/mL (Figure 2), our data indicate that the ratio of MBC to MIC is equal to 1; thus, we conclude that the *C. purpureus* exudate displays a bactericidal mode of action.

### 3.4. Light Sensitivity of Antibacterial Compounds Present in C. purpureus Exudates

**Figure 5:**
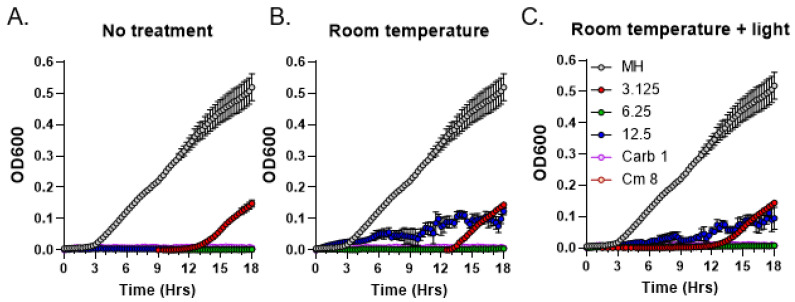
Residual antibacterial activity of *C. purpureus* R40 exudates after different treatments. Residual activity of exudate from four-week-old *C. purpureus* R40 culture was tested after treatments by MIC assays against *S. aureus.* (**A**) Samples were used in the assay immediately, without keeping them for 3 h at room temperature (untreated control). Samples were also kept at room temperature and either protected from light (**B**) or exposed to direct sunlight (**C**). Experiments were performed using 3.125, 6.25, and 12.5 mg/mL exudate concentrations. Carbenicillin (Carb, 1 µg/mL) and chloramphenicol (Cm, 8 µg/mL) were used as positive controls. MH, *S. aureus* growth in liquid MH medium without exudate addition.

As the first step towards the characterization of the chemical nature of the moss metabolites with antibacterial activity, we tested bioactive compounds present in R40 exudates for stability at ambient temperature and for light sensitivity. Test tubes containing moss exudates were either covered in aluminum foil or exposed to direct sunlight for 3 h at room temperature and subsequently analyzed by MIC assays to test for any detrimental effects on antibacterial activity. Carbenicillin and chloramphenicol antibiotics in their MIC concentrations as established for the *S. aureus* strain were used as a positive control [20]. Neither treatment regimen changed MIC values of the exudates compared to the control with no treatment (Figure 5). These data suggest that the bioactive moss metabolites are not photo-sensitive and relatively stable at ambient temperature.

### 3.5. Size Fractionation of Bioactive C. purpureus Exudate Components

**Figure 6:**
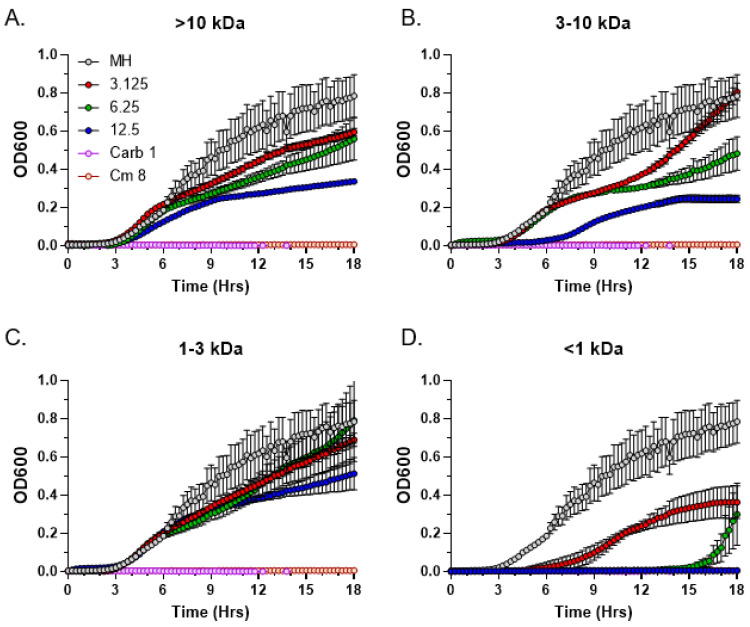
Size fractionation of *C. purpureus* exudates. Exudate from four-week-old *C. purpureus* R40 exudate was fractionated into four molecular weight fractions: >10 kDa (**A**), 3–10 kDa (**B**), 1–3 kDa (**C**), and <1 kDa (**D**). Each exudate fraction was analyzed by MIC assay at three different concentrations (3.125, 6.25, and 12.5 mg/mL) against *S. aureus* ATCC25923. MH medium without exudate addition was used as the negative control. Carbenicillin (Carb, 1 µg/mL) and chloramphenicol (Cm, 8 µg/mL) were used as positive controls.

To determine the approximate molecular weight range of the antimicrobial *C. purpureus* exudate compounds, we performed size fractionation using the Macrosep Advance centrifugal columns with molecular weight cutoffs of 10 kDa, 3 kDa, and 1 kDa. Following size fractionation, fractions were analyzed by the MIC assay. Antimicrobial activity was completely absent in three of the four size fractions: >10 kDa (Figure 6A), 3–10 kDa (Figure 6B), and 1–3 kDa (Figure 6C). In contrast, the <1 kDa fraction harbored substantial antimicrobial activity, with MIC values for the fractionated exudate being similar to the unfractionated R40 samples, 6.25 mg/mL (Figure 6D). We conclude that the molecular weight of the bioactive *C. purpureus* compounds is relatively low, with the maximum upper limit not exceeding 1 kDa.

### 3.6. Thermostability and Sensitivity to Proteinase K Treatment

**Figure 7:**
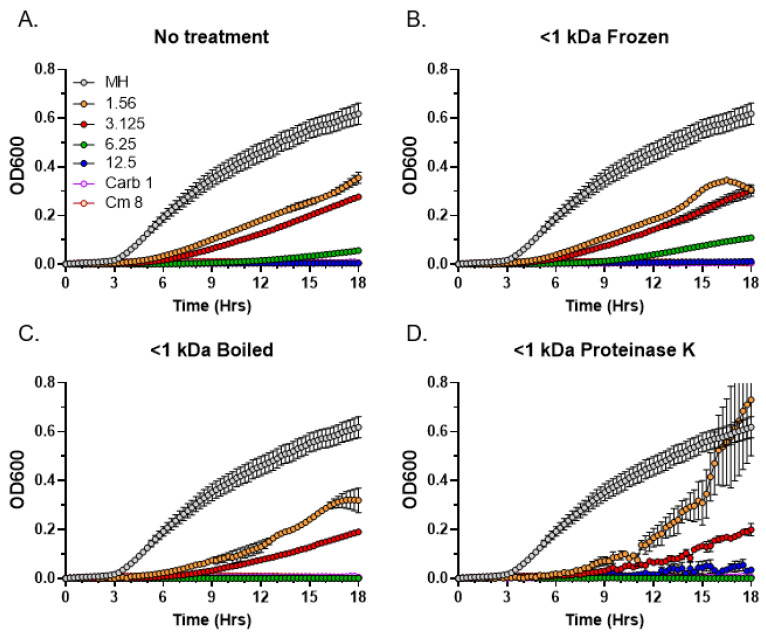
Residual antibacterial activity of *C. purpureus* R40 <1 kDa fraction after temperature and Proteinase K treatments. Residual activity of exudate from four-week-old *C. purpureus* R40 culture was tested after treatments by MIC assays against *S. aureus.* (**A**) Untreated control. (**B**–**D**) Samples were treated with repeated freezing and thawing (**B**), boiling (**C**) and incubation with Proteinase K (**D**). Samples were tested at exudate concentrations of 1.56, 3.125, 6.25, and 12.5 mg/mL. Carbenicillin (Carb, 1 µg/mL) and chloramphenicol (Cm, 8 µg/mL) were used as positive controls. MH, *S. aureus* growth in liquid MH medium without addition of exudate fraction.

After determining the approximate molecular weight range of the antibacterial *C. purpureus* R40 exudate components, we performed a series of thermostability and sensitivity to Proteinase K tests on the <1 kDa fraction containing the bioactive metabolites. Interestingly, similar to the untreated control (Figure 7A), antibacterial activity was not affected by repeated freezing and thawing cycles (Figure 7B) or by boiling (Figure 7C). Similarly, the exudate activity in the <1 kDa fraction was also not sensitive to the Proteinase K treatment (Figure 7D). These data indicate that the partially purified compounds are relatively thermostable and are unlikely to be proteinase-sensitive peptides or small proteins.

## 4. Discussion

Flowering plants exude a number of phytochemicals into the rhizosphere that can influence soil characteristics, inhibit or stimulate root interactions with microorganisms, and promote plant growth [32]. While mosses do not have roots, they also secrete a number of complex compounds and peptides into the environment, with potential functions in antimicrobial defense and immune signaling [19,33]. We have previously detected potent antimicrobial activity in exudates from the model moss *Physcomitrium patens* and characterized its activity against *Staphylococcus aureus* ATCC25923 and several other Gram-positive bacteria [20]. Here, we extended our search for antimicrobial metabolites from model mosses and analyzed exudates from the dioicous moss *Ceratodon purpureus*.

Exudates of the *C. purpureus* R40 strain displayed a range of antimicrobial activities against *S. aureus* and two other Gram-positive bacteria, *E. faecium* and *S. pyogenes*, which represent close relatives of GAS and VRE bacteria from the CDC “Biggest Threats” list (https://www.cdc.gov/drugresistance/biggest-threats.html, accessed on 30 January 2023). Specifically, R40 exudates showed relatively high activity against *S. aureus* and *E. faecium* but low activity against *S. pyogenes*. These data are intriguing as Staphylococci and Streptococci are both classified as Gram-positive, non-motile, non-sporing, and facultative anaerobic cocci, yet R40 exudates appear to be effective against Staphylococci but not so much against Streptococci. These differences in activity coupled with known structural variations in cell wall or bacterial physiology can be further explored to characterize the specific molecular mechanisms of exudate’s antibacterial action. Interestingly, the bioactive *C. purpureus* exudate compounds appear to have a bactericidal mode of action against *S. aureus* bacteria, as their MIC values are similar to the MBC values. As future research will focus on testing R40 exudates against a larger spectrum of Gram-positive pathogenic bacteria, a better understanding of their mode of antibacterial action will offer more meaningful prediction of their efficacy in vivo.

In contrast to the situation with Gram-positive bacteria, no antibacterial activity of *C. purpureus* exudates was observed against Gram-negative species *Salmonella* Typhimurium or *Serratia marcescens*. These data correlate well with our previous results on antibacterial activity of exudates from the moss *P. patens* [20] and suggest that the unique antimicrobial specificity towards Gram-positive bacteria is a unifying feature of exudates from two different model mosses that represent distinct subclasses of Bryopsida: Dicranidae (Ceratodon) and Funariidae (Physcomitrium). The biological significance of this observation remains to be established, but may involve implications for the type of bacteria mosses are often exposed to in their natural environment.

Several hundred different phytochemicals have previously been isolated from various mosses, with many of them possessing antimicrobial and antifungal activity [8,12]. However, most of these secondary plant metabolites have been detected in crude extracts of whole cells, making it more challenging to separate individual bioactive compounds from other components present in raw cellular mixtures. In contrast, exudates typically contain fewer components with more specific chemical structures that plants can easily secrete through the cell wall into the environment [14], making it potentially easier to isolate and further characterize the water-soluble bioactive compounds of interest. Furthermore, our time course data indicate that while antibacterial activity can be detected in R40 exudates as early as after 1 week of moss growth, it reaches maximum levels at four weeks, suggesting that the antimicrobial compounds are relatively stable in plant growth medium under standard conditions. This conclusion is further corroborated by our thermostability and light sensitivity assays, by apparent low molecular weight and by complete insensitivity to Proteinase K treatment. Collectively, these conclusions are encouraging, indicating that the chemical structure of *C. purpureus* R40 antibacterial compounds may be positively identified and characterized in the future.

Interestingly, only exudates from the male *C. purpureus* R40 strain, but not the female GG1 strain, exhibited strong activity against *S. aureus*, implying intriguing sexual dimorphism for the presence of metabolites with antibacterial activity in this dioicous moss species. The underlying mechanism of such dimorphism is currently unknown and will require identification of the chemical nature of the secreted metabolites from the male R40 strain. Nevertheless, sexual dimorphism in *C. purpureus* strains has previously been reported for a number of traits, with females having larger leaves and generally greater values for photosynthetic parameters [34]. Particularly compelling is the example of sex-specific compounds that influence moss fertilization and overall fitness. *C. purpureus* strains emit complex volatile scents, whose chemical composition and abundance are sex-specific, with moss-dwelling microarthropods being preferentially attracted to the female-produced volatile cues [24]. As the presence of sperm-dispersing microarthropods increases reproductive rates for *C. purpureus* moss strains [35], these data suggest that moss compounds released into the environment can indeed lead to a substantial fitness benefit. It would be similarly interesting to analyze the effects of sexual dimorphism in secreted antibacterial compounds on stress response, fitness, or physiological differences in *C. purpureus* male and female strains. Another implication of our study is that if genetic variation in secreted antibacterial compounds can also be demonstrated for *C. purpureus* male and female strains in their natural habitats, R40- and GG1-associated microbiomes could also be different, as was recently demonstrated for roots and rhizosphere soils of the dioecious flowering plant *Carica papaya* [36].

Further experiments, such as mass spectrometry analysis, will be necessary to establish both the nature of R40 antibacterial exudate components and their biological role in the *C. purpureus* life cycle. Currently, metabolomic, proteomics, and transcriptomics assays are well-established for *P. patens* [19,33,37,38], and similarly powerful tools are also being developed for *C. purpureus* [25]. Generally, the metabolome of *C. purpureus* appears to be diverse, with a number of biflavonoids, phospholipids, disaccharides, long-chain fatty acids, carotenoids, and antioxidants that change in abundance depending on the environmental conditions [26]. Some of the identified intracellular or cell-wall-bound biflavonoids display antioxidant and UV-protective activity [9]. However, little is known about natural metabolites secreted by *C. purpureus* into the environment. Future progress in characterizing secretome and exudate components will be instrumental in helping to identify new potent antimicrobial compounds from this model moss species.

## Figures and Tables

**Table 1:** Bacterial growth inhibition activity of *C. purpureus* exudates.

		Bacterial Growth Inhibition Zone in DDM Assays, in mm				MIC Values, mg/mL		MBC, mg/mL
*C. purpureus* strain	Bacteria	No Exudate Control ^a^	1-Week-Old Moss Exudate	2-Week-Old Moss Exudate	4-Week-Old Moss Exudate	2-Week-Old Moss Exudate	4-Week-Old Moss Exudate	4-Week-Old Moss Exudate
R40	*S. aureus*	7	10.00 ± 1.41 *	9.31 ± 1.09 **	15.71 ± 0.95 **	12.5	6.25	6.25
	*S. pyogenes*	7	10.67 ± 1.16 *	8.00 ± 1.00	13.75 ± 2.17 **	-	50	-
	*E. faecium*	7	8.50 ± 1.32	11.00 ± 0.01 **	14.88 ± 1.05 **	-	12.5	-
	*S. marcescens*	7	7	7	7	-	-	-
	*S. enterica* ser. Typhimurium	7	7	7	7	-	-	-
GG1	*S. aureus*	7	7	7	7	50	50	-
	*S. marcescens*	7	7	7	7	-	-	-
	*S. enterica* ser. Typhimurium	7	7	7	7	-	-	-

^a^ Cellulose disk diameter is 7 mm (no antibacterial activity). * Statistical significance *p* ≤ 0.05; ** statistical significance *p* ≤ 0.01; - not determined.

## Data Availability

The authors confirm that the data supporting the findings of this study are available within the article.

## References

[1] GBD 2019 Antimicrobial Resistance Collaborators (2022). Global mortality associated with 33 bacterial pathogens in 2019: A systematic analysis for the Global Burden of Disease Study 2019. Lancet.

[2] Patel J., Harant A., Fernandes G., Mwamelo A.J., Hein W., Dekker D., Sridhar D. (2023). Measuring the global response to antimicrobial resistance, 2020–2021: A systematic governance analysis of 114 countries. Lancet Infect. Dis..

[3] Antimicrobial Resistance Collaborators (2022). Global burden of bacterial antimicrobial resistance in 2019: A systematic analysis. Lancet.

[4] Asokan G.V., Ramadhan T., Ahmed E., Sanad H. (2019). WHO Global Priority Pathogens List: A Bibliometric Analysis of Medline-PubMed for Knowledge Mobilization to Infection Prevention and Control Practices in Bahrain. Oman Med. J..

[5] Mattingly J.M., Dunham C.M. (2022). ESKAPE velocity: Total synthesis platforms promise to increase the pace and diversity of antibiotic development. Nat. Struct. Mol. Biol..

[6] Khameneh B., Iranshahy M., Soheili V., Bazzaz B.S.F. (2019). Review on plant antimicrobials: A mechanistic viewpoint. Antimicrobial Resistance and Infection Control. BMC.

[7] Shen B. (2015). A New Golden Age of Natural Products Drug Discovery. Cell.

[8] Horn A., Pascal A., Lončarević I., Marques R., Lu Y., Miguel S., Bourgaud F., Thorsteinsdóttir M., Cronberg N., Becker J.D. (2021). Natural Products from Bryophytes: From Basic Biology to Biotechnological Applications. Crit. Rev. Plant Sci..

[9] Waterman M.J., Nugraha A.S., Hendra R., Ball G.E., Robinson S.A., Keller P.A. (2017). Antarctic Moss Biflavonoids Show High Antioxidant and Ultraviolet-Screening Activity. J. Nat. Prod..

[10] Kang S.J., Kim S.H., Liu P., Jovel E., Towers G.H. (2007). Antibacterial activities of some mosses including *Hylocomium splendens* from South Western British Columbia. Fitoterapia.

[11] Wolski G.J., Sadowska B., Fol M., Podsędek A., Kajszczak D., Kobylińska A. (2021). Cytotoxicity, antimicrobial and antioxidant activities of mosses obtained from open habitats. PLoS ONE.

[12] Olofin T.A., Akande A.O., Oyetayo V.O. (2013). Assessment of the antimicrobial properties of fractions obtained from bryophytes. J. Microbiol. Antimicrob..

[13] Mishra R., Pandey V.K., Chandra R. (2014). Potential of Bryophytes as therapeutics. IJPSR.

[14] Seitz V.A., McGivern B.B., Daly R.A., Chaparro J.M., Borton M.A., Sheflin A.M., Kresovich S., Shields L., Schipanski M.E., Wrighton K.C. (2022). Variation in Root Exudate Composition Influences Soil Microbiome Membership and Function. Appl. Environ. Microbiol..

[15] Hu L., Robert C.A.M., Cadot S., Zhang X., Ye M., Li B., Manzo D., Chervet N., Steinger T., van der Heijden M.G.A. (2022). Root exudate metabolites drive plant-soil feedbacks on growth and defense by shaping the rhizosphere microbiota. Nat. Comm..

[16] Samaddar S., Karp D.S., Schmidt R., Devarajan N., McGarvey J.A., Pires A.F.A., Scow K. (2021). Role of soil in the regulation of human and plant pathogens: Soils’ contributions to people. Phil. Trans. R. Soc. B.

[17] Ichino T., Yazaki K. (2022). Modes of secretion of plant lipophilic metabolites via ABCG transporter-dependent transport and vesicle-mediated trafficking. Curr. Opin. Plant Biol..

[18] Romani F., Banić E., Florent S.N., Kanazawa T., Goodger J.Q.D., Mentink R.A., Dierschke T., Zachgo S., Ueda T., Bowman J.L. (2020). Oil body formation in *Marchantia polymorpha* is controlled by MpC1HDZ and serves as a defense against arthropod herbivores. Curr. Biol..

[19] Fesenko I., Azarkina R., Kirov I., Kniazev A., Filippova A., Grafskaia E., Lazarev V., Zgoda V., Butenko I., Bukato O. (2019). Phytohormone treatment induces generation of cryptic peptides with antimicrobial activity in the Moss *Physcomitrella Patens*. BMC Plant Biol..

[20] Valeeva L.R., Dague A.L., Hall M.H., Tikhonova A.E., Sharipova M.R., Valentovic M.A., Bogomolnaya L.M., Shakirov E.V. (2022). Antimicrobial Activities of Secondary Metabolites from Model Mosses. Antibiotics.

[21] Biersma E.M., Convey P., Wyber R., Robinson S.A., Dowton M., van de Vijver B., Linse K., Griffiths H., Jackson J.A. (2020). Latitudinal Biogeographic Structuring in the Globally Distributed Moss *Ceratodon Purpureus*. Front. Plant Sci..

[22] Carey S.B., Jenkins J., Lovell J.T., Maumus F., Sreedasyam A., Payton A.C., Shu S., Tiley G.P., Fernandez-Pozo N., Healey A. (2021). Gene-rich UV sex chromosomes harbor conserved regulators of sexual development. Sci. Adv..

[23] Kollar L.M., Kiel S., James A.J., Carnley C.T., Scola D.N., Clark T.N., Khanal T., Rosenstiel T.N., Gall E.T., Grieshop K. (2021). The genetic architecture of sexual dimorphism in the moss *Ceratodon purpureus*. Proc. Biol. Sci..

[24] Rosenstiel T.N., Shortlidge E.E., Melnychenko A.N., Pankow J.F., Eppley S.M. (2012). Sex-specific volatile compounds influence microarthropod-mediated fertilization of moss. Nature.

[25] Brennan D.L., Kollar L.M., Kiel S., Deakova T., Laguerre A., McDaniel S.F., Eppley S.M., Gall E.T., Rosenstiel T.N. (2022). Measuring volatile emissions from moss gametophytes: A review of methodologies and new applications. Appl. Plant Sci..

[26] Sala-Carvalho W.R., Montessi-Amaral F.P., Esposito M.P., Campestrini R., Rossi M., Peralta D.F., Furlan C.M. (2022). Metabolome of *Ceratodon purpureus* (Hedw.) Brid.; a cosmopolitan moss: The influence of seasonality. Planta.

[27] Ashton N.V., Cove D.J. (1977). The Isolation and Preliminary Characterization of Auxotrophic and Analogue Resistant Mutants of the Moss, *Physcomitrella patens*. Molec. Gen. Genet..

[28] Shirshikova T.V., Sierra-Bakhshi C.G., Kamaletdinova L.K., Matrosova L.E., Khabipova N.N., Evtugyn V.G., Khilyas I.V., Danilova I.V., Mardanova A.M., Sharipova M.R. (2021). The ABC-Type Efflux Pump MacAB is Involved in Protection of *Serratia marcescens* against Aminoglycoside Antibiotics, Polymyxins, and Oxidative Stress. mSphere.

[29] Pankey G.A., Sabath L.D. (2004). Clinical relevance of bacteriostatic versus bactericidal mechanisms of action in the treatment of Gram-positive bacterial infections. Clin. Infect. Dis..

[30] Wald-Dicker N., Holtom P., Spellberg B. (2018). Busting the myth of “static vs. cidal”: A systemic literature review. Clin. Infect. Dis..

[31] Motyl M., Dorso K., Barrett J., Giacobbe R. (2003). Basic Microbiological Techniques for Antibacterial Drug Discovery. Curr. Protoc. Pharmacol..

[32] Badri D.V., Vivanco J.M. (2009). Regulation and function of root exudates. Plant Cell Environ..

[33] Lyapina I., Filippova A., Kovalchuk S., Ziganshin R., Mamaeva A., Lazarev V., Latsis I., Mikhalchik E., Panasenko O., Ivanov O. (2021). Possible role of small secreted peptides (SSPs) in immune signaling in bryophytes. Plant Mol. Biol..

[34] Slate M.L., Rosenstiel T.N., Eppley S.M. (2017). Sex-specific morphological and physiological differences in the moss *Ceratodon purpureus* (Dicranales). Ann. Bot..

[35] Shortlidge E.E., Carey S.B., Payton A.C., McDaniel S.F., Rosenstiel T.N., Eppley S.M. (2021). Microarthropod contributions to fitness variation in the common moss *Ceratodon purpureus*. Proc. Biol. Sci..

[36] Zhou Y., Pang Z., Yuan Z., Fallah N., Jia H., Ming R. (2022). Sex-based metabolic and microbiota differences in roots and rhizosphere soils of dioecious papaya (*Carica papaya* L.). Front. Plant Sci..

[37] Fesenko I., Shabalina S.A., Mamaeva A., Knyazev A., Glushkevich A., Lyapina I., Ziganshin R., Kovalchuk S., Kharlampieva D., Lazarev V. (2021). A vast pool of lineage-specific microproteins encoded by long non-coding RNAs in plants. Nucleic Acids Res..

[38] Fesenko I., Khazigaleeva R., Kirov I., Kniazev A., Glushenko O., Babalyan K., Arapidi G., Shashkova T., Butenko I., Zgoda V. (2017). Alternative splicing shapes transcriptome but not proteome diversity in *Physcomitrella patens*. Sci. Rep..

